# High-Performance Genome Annotation for a Safer and Faster-Developing Phage Therapy

**DOI:** 10.3390/v17030314

**Published:** 2025-02-25

**Authors:** Antoine Culot, Guillaume Abriat, Kieran P. Furlong

**Affiliations:** 1 Rime Bioinformatics SAS, 99120 Palaiseau, France; guillaume.abriat@rime-bioinformatics.com; 2 Department of Biochemistry, Microbiology and Immunology, Faculty of Medicine, University of Ottawa, Ottawa, ON K1H8M5, Canada

**Keywords:** SEA-PHAGE, rTOOLS, phage, bioinformatics, phage therapy, annotation

## Abstract

Phage therapy, which uses phages to decrease bacterial load in an ecosystem, introduces a multitude of gene copies (bacterial and phage) into said ecosystem. While it is widely accepted that phages have a significant impact on ecology, the mechanisms underlying their impact are not well understood. It is therefore paramount to understand what is released in the said ecosystem, to avoid alterations with difficult-to-predict—but potentially huge—consequences. An in-depth annotation of therapeutic phage genomes is therefore essential. Currently, the average published phage genome has only 20–30% functionally annotated genes, which represents a hurdle to overcome to deliver safe phage therapy, for both patients and the environment. This study aims to compare the effectiveness of manual versus automated phage genome annotation methods. Twenty-seven phage genomes were annotated using SEA-PHAGE and Rime Bioinformatics protocols. The structural (gene calling) and functional annotation results were compared. The results suggest that during the structural annotation step, the SEA-PHAGE method was able to identify an average of 1.5 more genes per phage (typically a frameshift gene) and 5.3 gene start sites per phage. Despite this difference, the impact on functional annotation appeared to be limited: on average, 1.2 genes per phage had erroneous functions, caused by the structural annotation. Rime Bioinformatics’ tool (rTOOLS, v2) performed better at assigning functions, especially where the SEA-PHAGE methods assigned hypothetical proteins: 7.0 genes per phage had a better functional annotation on average, compared to SEA PHAGE’s 1.7. The method comparison detailed in this article indicate that (1) manual structural annotation is marginally superior to rTOOLS automated structural annotation; (2) rTOOLS automated functional annotation is superior to manual functional annotation. Previously, the only way to obtain a high-quality annotation was by using manual protocols, such as SEA-PHAGES. In the relatively new field of phage therapy, which requires support to advance, manual work can be problematic due to its high cost. Rime Bioinformatics’ rTOOLS software allows for time and money to be saved by providing high-quality genome annotations that are comparable to manual results, enabling a safer and faster-developing phage therapy.

## 1. Introduction

### 1.1. Importance and Challenges of Genomics for Phage Therapy

#### 1.1.1. Higher Standards in Genomics Are Key for the Safe Use of Phage Therapy

Bacteriophages (phages) are bacterial viruses that are increasingly perceived as a promising solution to fight Antimicrobial Resistance (AMR) [1]. However, not all phages are good candidates for healthcare. Some can transfer genes between bacteria, making the bacteria more harmful [2,3]. For instance, if a phage carrying disease-causing toxin genes, as illustrated in the *Vibrio cholerae* example [4], was used in therapy, then the phage could cause harm. Further, if a phage is used in agriculture, then its genes will spread in the environment. If the phage is not well characterized and its genes are not understood, then this could lead to unpredictable environmental consequences [5,6,7].

The phage therapy community is very aware of this risk, as demonstrated by the published regulations and guidelines [8,9,10]. It is therefore crucial to evaluate the genes of every therapeutic phage candidate. The only time-efficient way to accomplish this is through phage genome annotation using bioinformatics to characterize as many phage genes as possible. The quality of such annotation is also limited by the assembly, which needs to be a reliable representation of the phage’s genomic sequence.

Currently, the average published phage genome has only 20–30% functionally annotated genes, which represents a hurdle to overcome to deliver safe phage therapy, for both patients and the environment [11]. Shallow genome annotation also hampers basic research, as it reduces a researcher’s understanding of a given phage. The poor quality of phage genomics and the lack of tools for the community have been noted by several authors [12,13,14,15]. However, the issue of quality in bioinformatics goes beyond the phage community. As Steinegger et al. discovered, even some versions of the world-widely used human reference genome are contaminated by foreign sequences [16,17,18].

**Figure 1:**
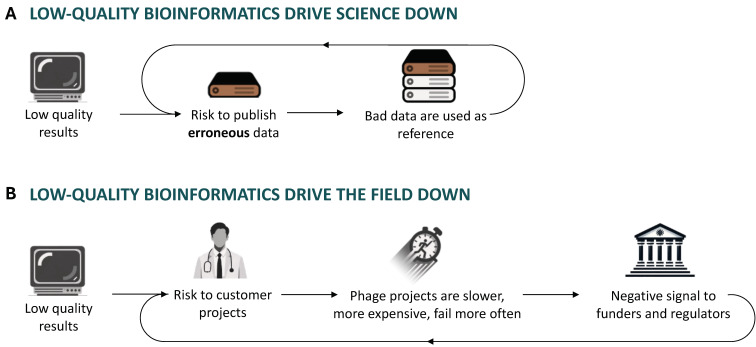
Impact of bioinformatics research quality on science and the phage therapy field. (**A**) A poorly annotated gene is published in an online database and re-used by the next researcher: a vicious cycle of database poisoning starts. (**B**) A poor bioinformatics analysis is used to assess a phage’s safety for phage therapy. Phages with a phage-therapy-incompatible gene content pass the bioinformatics screening and cause budget over-runs, delays, or project failure. This is reported to funders and regulators, who stop investing in the phage therapy field. In time, fewer funds are available for the next projects, which increases failure rates.

The quality of scientific publication and peer review is a growing concern in scientific communities [19,20,21], and the quality of bioinformatics data is also a concern. As every research finding is built on the foundations laid by previous works, published erroneous findings can mislead several subsequent scientists, who might cite and spread them. This phenomenon is in principle mitigated by peer review, which is completely absent of the genome publishing process in a database like NCBI Nucleotide or ENA EMBL (Figure 1A).

Li and Shen noted that Computer Science and Clinical and Life Sciences are the two fields that suffer from the most article retraction for academic misconduct [22]. As the current article’s topic is at the interface of both fields, it is the authors’ duty to encourage readers to exercise critical thinking as they read through this paper.

#### 1.1.2. Bad Genomics Will Slow Down Phage Therapy Development 

Phage therapy is (again) a relatively new topic in the West [23] and involves many players: academic research labs, private companies of all sizes, regulators, and funders. Academia is the main producer of new knowledge, which companies valorize to develop technology for end users. As companies make more “phage technology”, the user base grows, which in turn creates a market that attracts investors, which leads to more funding for those companies and academic research. This results in a virtuous cycle which promotes academic research, economic growth, public health, and environment preservation. Poor research practices increase the risk of failure for industrial projects, which generally happens during clinical trials performed by medium-sized startup companies. Clinical trial failure often means bankruptcy for startup companies, and funders lose their investment. As phage therapy is a small field, news spreads fast, and funders, contemplating an investment, are discouraged from taking part in the field’s growth. With fewer financial resources, the private market shrinks, reduces its investment in academic research, and a vicious, technology-delaying cycle starts (Figure 1B). The main victims of this cycle are likely to be the predicted 10,000,000 yearly deaths caused by drug-resistant bacteria by 2050 [7]. This mechanism is common to all critical R&D steps, including bioinformatics. In phage therapy research and development, favoring quantity at the expense of required quality could, therefore, be a costly mistake for mankind.

### 1.2. Efforts to Solve the Challenges of Phage Genomics

#### 1.2.1. Why Are We Facing These Challenges?

**Peer review**. Biology research requires skills in chemistry, physics, and math, as well as mastering a wide array of diverse wet lab techniques. Math and computer science might not be biologists’ strong points [24]; little importance is usually given to bioinformatics in the “Materials and Methods” sections of microbiology papers. It is indeed common to give temperatures for a PCR and the model and manufacturer of an electron microscope, but it is very rare to find the version and options used for a bioinformatics tool. This hampers reproducibility, and therefore peer review, which is at the core of the modern academic system.

**Bioinformatics makes use of user-unfriendly interfaces**, such as the command line. Most biologists are not familiar with those tools, which makes them harder to use, and inevitably drives scientific results down.

**Trained bioinformaticians are scarce**. Most research teams do not have the funds or enough workload for a dedicated bioinformatician. Some bigger teams do. They share a bioinformatician to support their efforts in diverse research topics, which leaves little opportunity for that person to specialize and excel in a given field. Working with a trained phage bioinformatician is a privilege few teams can experience.

**Price is the historical driver of the sequencing market**. In the 1990s, when the first human genome was being sequenced, this technology was much more expensive than it is nowadays [25]. It was therefore rarely used, until large companies found a way to drive costs down, using new technologies, standardization, and economy of scale. Big sequencing companies are now the largest providers of bioinformatics services, and they still use standardized, one-size-fits-all pipelines to work on genome data, no matter the studied organism. This low-price, high-quantity policy yields poor-quality results that are eventually published in scientific journals and databases.

#### 1.2.2. Promoting Better Phage Genomics

Despite the aforementioned issues, the phage genomics field is pushed forward by bioinformaticians and biologists who create free, ever more user-friendly, and innovative tools: any dedicated researcher can have access to Pharokka [26], CPT’s Galaxy [27], or study a protein’s 3D structure with Colabfold [28]. High-level training is also available, for instance to students who take part in the SEA-PHAGES (Science Education Alliance-Phage Hunters Advancing Genomics and Evolutionary Science) program [29].

The program is led by Graham Hatfull’s group at the University of Pittsburgh and the Howard Hughes Medical Institute’s Science Education division and focuses on increasing undergraduate interest and retention in biological sciences. It achieves this by immersing students in discovering, characterizing, and naming their bacteriophages. More specifically, SEA-PHAGES students are presented with the opportunity to manually annotate phage genomes. For each genome, several young researchers and an experienced teacher study feature-by-feature the information encoded in the nucleotide sequence; this is the golden standard of bioinformatics phage genome annotation.

By 2019, 3000 phages were already sequenced, and 500 phages are sequenced every year [30]. The program has expanded to multiple universities in the US, Canada, and recently to France [31]. SEA-PHAGE’s methodology is tailored to produce high-quality results but requires a significant time investment, which makes it unfit for an industrial field such as phage therapy.

As academia is already significantly invested in pushing better practices in academic bioinformatics, what else can be done to promote faster and safer phage therapy?

#### 1.2.3. A New Project to Take Part in Academic Efforts to Enhance Phage Genomics

As discussed above, the successful widespread adoption of phage therapy depends on private and public research synergy. In particular, large industrial phage companies need to be provided with technological expertise and support, which is not the main mission of academia.

Rime Bioinformatics is a project which aims to promote better practices in bioinformatics, to provide faster and safer phage therapy for patients and the environment. The project has several components:

- High-performance bioinformatics software development;
- A combination of phage therapy, genomics, and regulation expertise;
- A focus on developing methods following strict quality standards (ISO 9001, GLP, GMP [32,33,34]) to ensure repeatable and reliable high performance.
- A strong engagement towards our mission:
  ○ Promotion of the advent of a safe phage therapy for patients and the environment;
  ○ Providing help to academia and industry with their academic projects;
  ○ Participating in global efforts to build the phage therapy ecosystem.

The road to implementing an impactful and self-originated scientific project generally undergoes several post-docs, years of academic competition, and the foundation of a new academic lab [35]. As Rime Bioinformatics’ mission requires close collaboration with academia and industry, and the time-to-impact is shorter for private companies, the project’s chosen legal status was a private company.

A company’s faster impact can, however, be mitigated by private-business-specific constraints: healthy companies are funded by customers, which pay the company to obtain access to its intellectual property (IP). A private company’s core IP is therefore necessarily protected, which prevents the unrestrained release of materials and methods and, therefore, of knowledge. Companies are also rightfully criticized for being subject to scientific bias, as their decisions are incentivized by customers’ and investor’s money. As academic researchers are also incentivized by grant-givers’ money to publish more—and not better—academic research’s probity is also negatively influenced by funding. Eventually, private and academic scientists come from the same amphitheaters, and intellectual honesty is individual-dependent.

### 1.3. The RimeTOOLS Pipeline for Phage Genome Annotation

#### 1.3.1. Using Large, Curated Databases to Produce High-Quality Annotations

Modern bioinformatics gene function annotation relies on a simple algorithm:

1. Finding a gene or domain that was already annotated using reliable methods.
2. Comparing the gene to annotate the already known gene or domain.
3. Deciding if the two compared genes are close enough to be considered functional homologs.
4. Repeat until success or failure.

This method is generally performed using gene databases. As a researcher is selecting a reference database, the choice is twofold:

- Using a small, high-quality, manually curated database like Swiss-Prot [36];
- Using a large, low-quality, uncurated database like NCBI Nucleotide [37].

Small, curated databases will yield reliable annotations, but the chances of finding a functional homolog to the gene to annotate are slim due to the small number of entries in the database. Large, uncurated databases will yield much more, but often unreliable, annotations.

Rime Bioinformatics’ pipeline (RimeTOOLS or rTOOLS) is based on publicly available tools, used to query public databases, which were cleaned using a proprietary database cleaner. The principle is to use large, curated databases to provide high-quality annotations. This large data input enables the pipeline to produce more reliable annotations. Stringent parameters are used to set each annotation tool: it is preferable to avoid giving a function to a gene rather than producing false positive results, which might contaminate the public database if published.

The pipeline can be fed with any type of gene function database and therefore adapted to prophage, phageome annotation, discovery, in-depth phage genome annotation, high-throughput characterization of a phage bank, and unknown gene function prediction. As it is primarily aimed at phage therapy research, rTOOLS is ISO9001:2015- and GxP-compliant. rTOOLS output files are also compliant with NCBI’s best practices for gene annotation [38].

**Figure 2:**
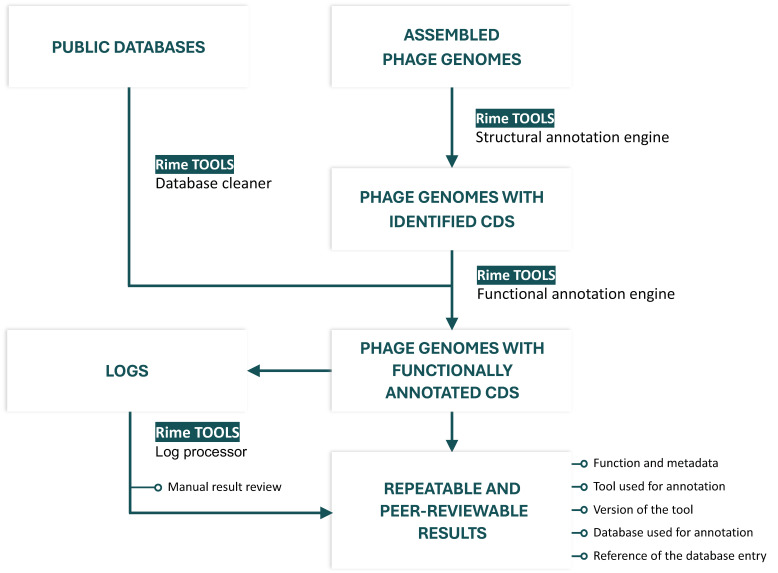
The RimeTOOLS pipeline for phage genome annotation.

The tools and data used for annotation are given with every result, which makes the results peer-reviewable: anyone with access to a computer and an internet connection can download the database entry and the software used to produce every annotation proposed by the pipeline (Figure 2).

#### 1.3.2. RimeTOOLS Is a Semi-Automatic Pipeline

High-throughput, unsupervised automatic analysis allows for the processing of huge datasets but is not resilient to new or unpredicted biological phenomena. rTOOLS is built to output what it does at every step, using detailed logs. The logs are then processed automatically to detect and remove inconsistent outputs, and to facilitate manual reviewing of all the annotations and the decision-making process of the pipeline. Depending on the use case, rTOOLS can be used in automatic or semi-automatic mode: large metagenomics datasets are, for example, not suitable for in-depth manual annotation review because of their size. In automatic mode, rTOOLS picks the best annotations automatically, whereas semi-automatic mode enables a manual selection and curation of all the results.

### 1.4. Benchmarking RimeTOOLS Versus SEA-PHAGES

The improvement in bioinformatics tools like rTOOLS raises the following question: “Can automatic annotation tools compare to manual approaches?”. This study aims to compare the phage genome annotation results obtained by the two methods to answer that question.

## 2. Methods

### 2.1. Phage Sequences

The phages in this study were discovered, and isolated, and their DNA was extracted following the SEA-PHAGES discovery guide [39]. Subsequently, the phage DNA was sequenced by preparing a sequencing library, using the NEB Ultra II FS kit, and sequencing using an Illumina-MiSeq instrument Illumina, Inc., San Diegeo, CA, USA. Following Russell [40], the raw reads were assembled using Newbler v.2.9 [41], which resulted in a single contig. The assembly was checked for completeness, accuracy, and genome termini using Consed v.29.0 [42]. The repository for the phages is located on the PhagesDB website [43].

### 2.2. Phage Genome Annotation

The contigs were annotated using the SEA-PHAGES protocol [44] and annotated in parallel using the RimeTOOLS2 Pipeline, from Rime Bioinformatics. Coding sequences (CDS) and tRNAs were predicted using the following tools: (Prodigal 2.6.3, PHANOTATE 1.5.0, Glimmer 3.02, GeneMarkS 1.14, tRNA-Scan-SE 2.0.7). CDS were subsequently used to query curated versions of public gene and protein databases (CARD 2023.05, ResFinder 2023.04, VFDB 2023.05, PHANTOME 2021.03, Swiss-Prot 2023.05, NCBI Virus 2023.15, VOG 2020.05, pVOG 2021.03, RefSeq 2023.05, DefenseFinder 2023.05, and PHROGs 2023.05 (6–13)) using HMMER 3.3.2, HHsuite 3.3.2, and Blast 2.9.0+. Results with high match scores were kept for the final functional annotation (BLAST+: E-value < 0.00001, Identity > 0.7, Coverage > 0.8; HHSUITE: E-value < 0.00001, Coverage > 0.7, Probability > 90%, Score > 30; HMMER: E-value < 0.00001, Coverage > 0.7). The pipeline was used in automatic mode.

### 2.3. Genome Annotation Comparison

This study looked at 27 phage genomes, comparing their automated annotations, using Rime Bioinformatics, to their manual annotations, using the SEA-PHAGES protocol. The SEA-PHAGE annotations were downloaded from NCBI GenBank [45]. A spreadsheet was then created for each of the phages to compare the gene start sites, stop sites, and gene functions of the automated and manual annotations (Supplementary Materials). The differences were then noted, and the SEA-PHAGES protocol was used to determine which annotation was considered better or if they were similar. For example, the function of phage Amyev’s gene 8 is annotated as “head-to-tail adapter” by SEA-PHAGES, which is similar to the RimeTOOLS annotation “head-to-tail adapter Ad1”.

## 3. Results and Discussion

### 3.1. Comparing Manual and Automated Annotation

The study of phage genomes plays an important role in academic research and phage therapeutic development. The SEA-PHAGES protocol for manual phage genome annotation represents the gold standard, providing the best results obtainable in silico. However, this protocol is time-consuming, as it requires several researchers with expertise, and is subject to human bias. Rime Bioinformatics’ protocol is fully automated and aims to match or supersede manual phage genome annotation. This approach offers faster annotation and reduces errors due to human bias. Nevertheless, it is challenging to implement a human reviewer’s knowledge and finesse. This study aims to compare the effectiveness of manual versus automated phage genome annotation methods. Twenty-seven phage genomes were annotated using SEA-PHAGES and Rime Bioinformatics protocols. The structural (gene calling) and functional annotation results were compared (comparison table available in the Supplementary Materials).

On average, 1.5 additional genes per phage and 5.3 gene start sites per phage were better identified by SEA-PHAGES methods at the structural annotation step. Despite this difference, the impact on functional annotation was limited: on average, 1.2 genes per phage had erroneous functions, caused by the structural annotation. The Rime Bioinformatics’ tool performed better at assigning functions, especially where the SEA-PHAGES methods assigned hypothetical proteins: 7.0 genes per phage had better functional annotation on average, compared to SEA PHAGE’s 1.7.

It is important to note that these results cannot be generalized to all phages: phage diversity is largely unknown, and the dataset used for the comparison is made up of *Arthrobacter* spp. phages, which induces a taxonomic bias.

While manual structural annotation remains superior to automated structural annotation, it has a limited impact on real-world applications: gene functions are the most valuable piece of information for most academic and industrial cases. To achieve the best results, both approaches should be combined, and this is why rTOOLS is designed to allow for manual revision at every step of the analysis.

### 3.2. In Silico and Wet Lab Annotation

It is important to note that certain discrepancies in annotations necessitate further wet lab experiments. For instance, limited wet lab experiments have demonstrated that when two tandem start sites exist, the second start site is typically selected (unpublished SEA-PHAGES data).

However, a question arises when two tandem start sites with a −1 and +2 gap are discovered, as seen in phage Liebe gene 59. The dilemma is whether to select the start with a −1 gap, which is preferred as the ribosome can reinitiate translation without disassembling and reassembling [46]. Or, if the start site with a +2 gap is more desirable since it is favored by some mass spectrometry data [47]. Alternatively, both start codons may be translated a certain percentage of the time.

In the case of the Liebe gene 59 annotation, the SEA-PHAGES annotation identifies the start site with a −1 gap, while the automated Rime Bioinformatics annotation identifies the start site with a −16 gap, which has a longer open reading frame (ORF), and which is often preferred.

If we prioritize mass spectrometry data, then both the manual and automated start site annotations for Liebe gene 59 are incorrect. This highlights that despite available documentation, user errors occur. This situation emphasizes the initial necessity for a dual approach, where manual annotation can serve to refine the automation process before wet lab validation for key genes. Over time, enhanced automation could supersede the need for manual annotations.

## 4. Conclusions

### 4.1. Academic Phage Genome Annotation

The method comparison detailed in this article demonstrated the following:

- Manual structural annotation is marginally superior to rTOOLS automated structural annotation.
- rTOOLS automated functional annotation is superior to manual functional annotation.

Phage genome annotation’s most challenging aspect is structural annotation. This step is crucial, as errors can significantly compromise functional annotation. If a substantial portion, or all, of a gene sequence is missed, it is more difficult or impossible to use that sequence to query gene function databases. To achieve high-quality results, manual annotation is necessary until software tools improve. In our results, the structural annotation difference was low enough not to impact functional annotation at the genome scale.

It is also important to remember that bioinformatics is a form of modeling and given the vast and largely unknown diversity of phages, the available models are incomplete. Supplementing bioinformatics with wet lab data is essential for obtaining truly reliable annotations.

### 4.2. Phage Genome Annotation for Phage Therapy

Using phages to decrease bacterial load in an ecosystem (a patient or the environment) is equivalent to releasing a large number of gene copies in this ecosystem. It is widely accepted that phages play a crucial ecological role, but the mechanisms governing this role are not well understood. It is therefore key to know what is released in the said ecosystem to avoid alterations with difficult-to-predict, but potentially huge, consequences. In-depth annotation of therapeutic phage genomes is therefore essential.

Previously, the only way to obtain such a high-quality annotation was by using manual protocols, such as SEA-PHAGES. In the relatively new field of phage therapy, which requires support to advance, manual work can be problematic due to its high cost. Rime Bioinformatics’ rTOOLS software allows for time and money to be saved by providing high-quality genome annotations that are comparable to manual results; this enables a safer and faster-developing phage therapy.

The phage community is currently laying foundations, which will be used as a reference for future phage therapy projects. If low—and unsafe—standards are accepted now, they will be the norm for future phage therapy projects, with significant consequences for patients and the environment. The precautionary principle dictates that we should prioritize safety to avoid future regret.

## Data Availability

Data are available in this article and in the supplementary data.

## Supplementary Materials

The following supporting information can be downloaded at: https://www.mdpi.com/article/10.3390/v17030314/s1, Spreadsheet: Comparison of the structural and annotation results from SEA-PHAGE and rTOOLS.
